# In Vitro DNA-Binding, Anti-Oxidant and Anticancer Activity of Indole-2-Carboxylic Acid Dinuclear Copper(II) Complexes

**DOI:** 10.3390/molecules22010171

**Published:** 2017-01-20

**Authors:** Xiangcong Wang, Maocai Yan, Qibao Wang, Huannan Wang, Zhengyang Wang, Jiayi Zhao, Jing Li, Zhen Zhang

**Affiliations:** School of Pharmacy, Jining Medical University, 669 Xueyuan Road, Rizhao 276800, Shandong, China; wxcwayne@126.com (X.W.); yanmaocai@126.com (M.Y.); 18263372608@163.com (Q.W.); wanghuannan0322@126.com (H.W.); 17806217108@163.com (Z.W.); 17806218007@163.com (J.Z.); 17862335612@163.com (J.L.)

**Keywords:** DNA-binding, antioxidant, anticancer, indole-2-carboxylicacid, copper(II) complexes

## Abstract

Indole-2-carboxylic acid copper complex (ICA-Cu) was successfully prepared and characterized through elemental analysis, IR, UV-Vis, ^1^H-NMR, TG analysis, and molar conductance, and its molecular formula was [Cu_2_(C_9_H_6_O_2_N)_4_(H_2_O)_2_]·2H_2_O. The binding ability of ICA-Cu to calf thymus DNA (CT-DNA) was examined by fluorescence spectrometry and the viscosity method. The results indicated that, upon the addition of increasing amounts of CT-DNA, the excitation and emission intensity of ICA-Cu decreased obviously and the excitation spectra shifted towards a long wavelength. ICA-Cu could displace ethidium bromide (EB) from the EB-DNA system, making the fluorescence intensity of the EB-DNA system decrease sharply; the quenching constant *K_SV_* value was 3.99 × 10^4^ M^−1^. The emission intensity of the ICA-Cu-DNA system was nearly constant, along with the addition of Na^+^ in a series of concentrations. The fluorescence of the complex could be protected after the complex interacted with DNA. A viscosity measurement further supported the result that the ICA-Cu complex may interact with DNA in an intercalative binding mode. The antioxidant activities of ICA-Cu were evaluated by a 2,2-diphenyl-1-picrylhydrazyl (DPPH) assay, a hydroxyl radical (OH) scavenging assay, and a 2,2′-azino-bis(3-ethylbenzothiazoline-6-sulphonic acid (ABTS) assay. The ICA-Cu exhibited the highest inhibitory effects on the ABTS radical (94% inhibition at 60 µM), followed by OH and DPPH radicals (the degrees of inhibition being 71% and 56%, respectively). The in vitro cytotoxicity activity of ICA-Cu against two human breast cancer cell lines, MDA-MB-231 and MCF-7, was investigated by 3-[4,5-dimethyltiazol2-yl]-2.5-diphenyl-tetrazolium bromide (MTT) assay and cellular morphological analysis. The results showed that, upon increasing the concentration of ICA-Cu, an increase was observed in growth-inhibitory activity and the inhibition percentage were greater than 90% at 20 µM in both cell lines. Also, cellular morphological changes in the two cell lines agreed with the cytotoxicity results.

## 1. Introduction

Now cancer has become the leading cause of death of the world, since the incidence of cancer is increasing every year [1,2]. Therefore, developing novel anticancer drugs has been a hot research region. As early as the 1960s, the development and clinical application of a platinum-based anticancer agent, cisplatin, had an enormous impact on cancer chemotherapy [3]. Cisplatin exerts its antitumor activities through binding to DNA strands, interfering with DNA replication and thereby triggering programmed cell death [4]. Although there are some limitations with cisplatin, including low solubility, significant toxicity, and resistance in some cancer types [5], cisplatin is still one of the most effective anticancer therapeutics against various solid tumors today, especially solid tumors of the head, ovary, melanoma, and lymphomas [6]. What is more, the effectiveness of cisplatin in cancer chemotherapy has stimulated people to investigate other metal complexes as potential antitumor agents. Current studies have demonstrated that a number of metal-based complexes, such as complexes of gold, gallium, copper, and manganese, possess promising inhibitory activities on various cancer cells and have been evaluated in vitro and in vivo in cancer therapy [7,8,9,10]. Milacic and Cattaruzza et al. [7,8] reported that dithiocarbamato gold(III) derivatives of amino acids have exhibited remarkable biological activities toward breast and prostate cancer cells, as well as their xenografts. Chen et al. [9] investigated the anticancer effects of gallium(III) complexes and found that they showed significant inhibitory activities against the tumor growth of human PC-3 xenografts on mice, which was associated with the inhibition of proteasome activity. More recently, we have studied a new manganese(II) complex that has exhibited potent antiproliferative activity on human breast cancer cells. Notably, the complex could inhibit proteasome activity and induce tumor cell apoptosis [10].

Copper (Cu) is one of the essential microelements in all living organisms, which acts as both a catalyst and as a part of enzymes [11,12]. In recent years, many studies have indicated that the Cu levels in serum and tissue are significantly greater in cancers of the breast, prostate, colon, lung, and brain, etc. [13,14,15,16]. Furthermore, Cu has been also demonstrated to be associated with angiogenesis [17], which is important for tumor proliferation, invasion, and metastasis [18]. Based on the biological function of Cu in tumor progression, a number of Cu complexes, including disulfiram (DSF)-Cu, clioquinol (CQ)-Cu, 8-hydroxyquinoline (8-OHQ)-Cu, pyrrolidine dithiocarbamate (PDTC)-Cu, diethyldithiocarbamate (DDTC)-Cu, and some Schiff base Cu complexes, were found to possess potent antitumor activities in vitro or in vivo [19,20,21,22,23].

**Figure 1:**
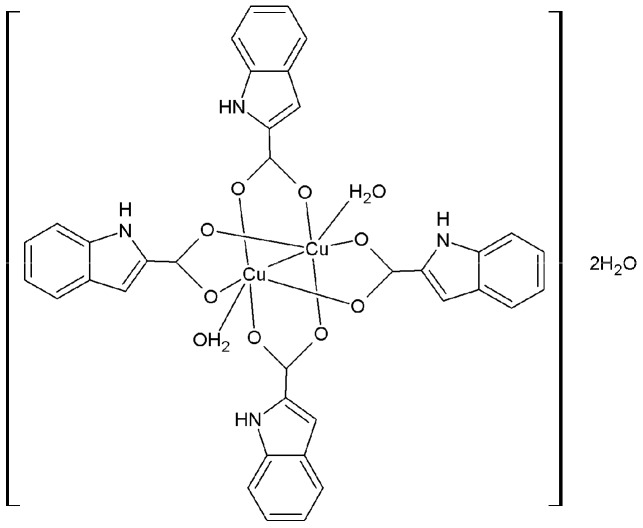
Chemical structure of the Indole-2-carboxylic acid copper complex (ICA-Cu).

Indoles are a class of heterocycles with important physiological functions and potent pharmacological activity, especially antitumor activity [24,25,26]. Our previous study results showed that the copper and cadmium complexes of indolecarboxylic acid could inhibit cell proliferation in human breast and prostate cancer cells, while showing lower toxicity to normal cells [27,28,29]. In the current research, the dinuclear copper(II) complex with indole-2-carboxylic acid (ICA) as the ligand has been synthesized, and the biological and anticancer properties of the indole-2-carboxylic acid copper(II) complex (ICA-Cu, Figure 1) have been examined. The binding ability of the complex to CT-DNA was studied by fluorescence spectrometry and the viscosity method, and we found that ICA-Cu possibly binds to DNA by the mode of intercalation. ICA-Cu showed high antioxidant activity in a DPPH assay, a hydroxyl radical scavenging assay, and an ABTS assay. In addition, the in vitro cytotoxicity of ICA-Cu on human breast cancer cells MDA-MB-231 and MCF-7 was evaluated by an MTT assay and cellular morphological analysis, and the results suggested that ICA-Cu possessed potent growth-inhibitory activity against the two cancer cell lines. Taken together, these results indicate that the complex ICA-Cu could be a promising lead compound for the development of anticancer drugs.

## 2. Results and Discussion

### 2.1. Synthesis and Characterization of ICA-Cu Complexes

We previously demonstrated that indole-3-carboxylic acid metal complexes exhibit potent anticancer activity on breast and prostate cancer cells. To further investigate such metal complexes and develop anticancer drug candidates, in this study indole-2-carboxylic acid copper complexes (ICA-Cu, Figure 1) were prepared and their structures were characterized through elemental analysis, IR, UV-Vis, ^1^H-NMR, TG analysis, and molar conductance. Moreover, DNA binding activity, antioxidant activity, and in vitro cytotoxicity were further investigated.

#### 2.1.1. Infrared Spectra

The IR absorptions and the assignments of the ICA-Cu complex are presented in Table 1. The complex exhibits a strong absorption peak at 3437.61 cm^−1^, due to the indole ring -NH- group. No significant shift was observed in comparison with the ligand, suggesting that the nitrogen in the indole ring did not coordinate with the copper ion. Compared to the ligand, the two peaks at 1510.92 cm^−1^ and 1344.34 cm^−1^ may be assigned to *υ_as_* (COO^−^) and *υ_s_* (COO^−^), respectively. Furthermore, the difference between *υ_as_* (COO^−^) and *υ_s_* (COO^−^) was less than 200 cm^−1^, which suggests that the oxygen of COO^−^ in the ligand is bound to Cu to form a bidentate complex [30]. A new absorption band appeared at 484.64 cm^−1^ in the complex, which could be attributed to the formation of the Cu-O bond.

#### 2.1.2. UV-Vis Absorption Spectra

UV-Vis absorption spectra of the ligand and the ICA-Cu complex were recorded in a 200–800 nm range. The λ_max_ values of the ligand and the complex appeared at 292 nm and 298 nm (Table 2), respectively, which was attributed to n-π* transitions. In addition, the absorption band was shifted to a longer wavelength, indicating the occurrence of a metal-to-ligand charge transfer transition.

#### 2.1.3. ^1^H-NMR Spectra

The complex was further characterized by ^1^H-NMR (DMSO-*d*_6_, 600 MHz, Table 3). Compared with the ligand (δ (ppm) 7.051 (m, H-5, indole ring); 7.238, (m, H-6, indole ring); 7.252, (m, H-3, indole ring); 7.442, (m, H-7, indole ring); 7.649, (m, H-4, indole ring); 11.753, (s, -COOH); and 12.943 (s, -NH-, indole ring)), the ^1^H-NMR spectra of ICA-Cu showed that there were small shifts in the signals, which were due to the redistribution of the ligand caused by the formation of metal complexes between the ligand and Cu(II). Furthermore, the signal of the -NH- proton appeared at 14.943 ppm (s, -NH-, indole ring), but the signal of the -COOH- proton disappeared in the spectra of the Cu(II) complex, indicating the coordination of the ligand to the copper through the oxygen of the -COO^−^ group.

#### 2.1.4. Thermogravimetric Analysis

Thermogravimetric (TG) analysis of ICA-Cu was performed in a temperature range from 25 to 800 °C. The complex decomposed mainly in two steps. A 4.34% (4.29%) weight loss was found at 25–120 °C, corresponding to the two moles of crystal water in the first step. In the second stage, the complex decomposed to give CuO. The residue rate of ICA-Cu was 19.75%, which was in agreement with the calculated value (19.05%).

#### 2.1.5. Elemental Analysis

The contents of C, H, and N in the ICA-Cu complex were determined by elemental analysis. The results agreed well with the theoretical formula of the complex.

#### 2.1.6. Molar Conductivity

The ICA-Cu complex was soluble in DMF and DMSO, slightly soluble in water and ethanol and stable in the air. The molar conductivity of ICA-Cu in DMSO was 16.52 S·cm^−1^·mol^−1^, which was less than 35 S·cm^−1^·mol^−1^ [31]. In consequence, the ICA-Cu could be identified as a nonelectrolyte and stable in solution.

### 2.2. DNA Binding Studies

#### 2.2.1. Fluorescence Spectroscopy Studies

**Figure 2:**
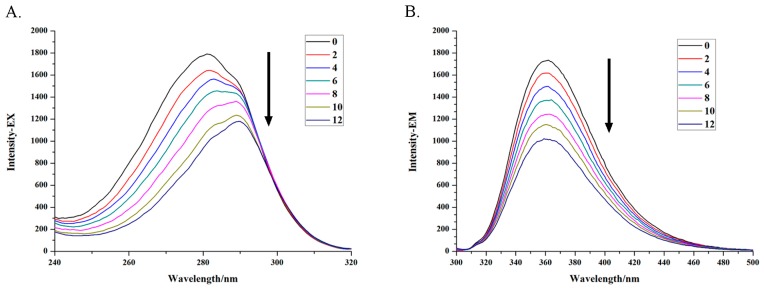
Excitation spectra (**A**) and emission spectra (**B**) of the ICA-Cu complex in the presence of increasing amounts of CT-DNA. The arrow indicates the fluorescence intensity changes of ICA-Cu upon increases of DNA concentration. r = 0, 2, 4, 6, 8, 10, 12 (r = [DNA]/[ICA-Cu]).

**Figure 3:**
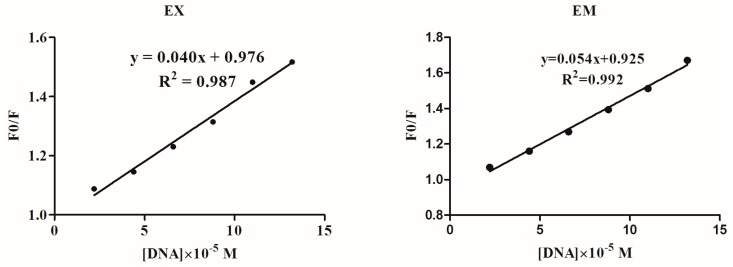
Plots of F_0_/F against [DNA] for the titration of the increasing concentration of DNA in the ICA-Cu complex solution.

The interaction of the complexes with DNA is very important for researching the mode of action of anticancer drugs. Herein, the DNA binding properties of ICA-Cu were firstly studied by fluorescence spectroscopy [32]. Increasing concentrations of DNA (2.2–13.2 × 10^−5^ M) were added to the fixed concentrations of ICA-Cu ([DNA]/[ICA-Cu] = 0, 2, 4, 6, 8, 10, 12) and the fluorescence excitation and emission spectra were recorded (Figure 2). It was clear from Figure 2 that ICA-Cu exhibited an emission band around 362 nm when excited at 282 nm. Upon the addition of increasing amounts of CT-DNA, the excitation and emission intensity of ICA-Cu decreased obviously (Figure 2). In addition, the excitation spectra shifted towards a long wavelength appreciably, as shown in Figure 2A. The significant quenching and red shift of the ICA-Cu fluorescence suggested that there was an interaction between the ICA-Cu and the DNA base pairs. The quenching efficiency could be calculated according to the linear Stern-Volmer equation [33], F_0_/F = 1 + *K_SV_* [Q]; here F_0_ and F indicate the fluorescence intensity in the absence or presence of a compound at [Q], respectively. *K_SV_* indicates the Stern-Volmer dynamic quenching constant. The plots of F_0_/F against [DNA] are linear curves (Figure 3) and the *K_SV_* values of excitation and emission spectra are 4.0 × 10^3^ M^−1^ and 5.4 × 10^3^ M^−1^, respectively. 

#### 2.2.2. Studies of Competitive DNA-Binding Ability with EB

**Figure 4:**
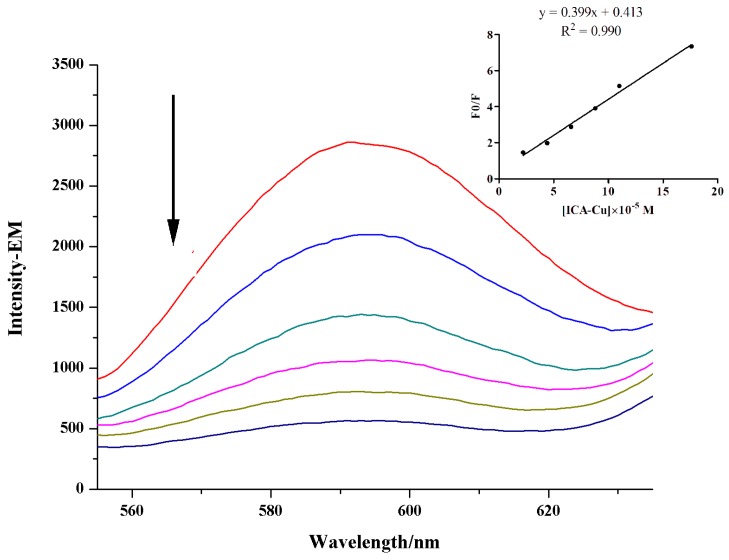
Emission spectra of the EB-DNA complex of fluorescence quenching with an increasing concentration of ICA-Cu and the arrow indicates the fluorescence intensity changes. Insert: plot of F_0_/F against [ICA-Cu]. r = 0, 2, 4, 6, 8, 10, 16 (r = [ICA-Cu]/[EB-DNA]).

Ethidium bromide (EB) is a fluorescent probe of DNA that has been used extensively in the study of binding mode of metal complexes to DNA [34]. In this study, DNA was incubated with EB and the concentration kept constant. The emission intensities of the EB-DNA system (emission band at 596 nm) were then measured along with the addition of the complex. As shown in Figure 4, the ICA-Cu complex could sharply decrease the fluorescence intensity of the EB-DNA system, and the fluorescence intensity almost disappeared after the ratio of [ICA-Cu]/[EB-DNA] was increased to 16:1. The remarkable quenching of the EB-DNA fluorescence indicated ICA-Cu could displace EB from the EB-DNA system, thus indicating that it interacts with DNA by an intercalative mode. The quenching constant was obtained by analyzing the data using the Stern-Volmer equation, in line with the method of fluorescence spectroscopy studies. The plot of F_0_/F against [ICA-Cu] was a linear curve (shown in Figure 4 inset). A relatively high *K_SV_* value of 3.99 × 10^4^ M^−1^ for the ICA-Cu further demonstrated that ICA-Cu could compete for DNA-binding sites with EB.

#### 2.2.3. Effect of Ionic Strength

**Figure 5:**
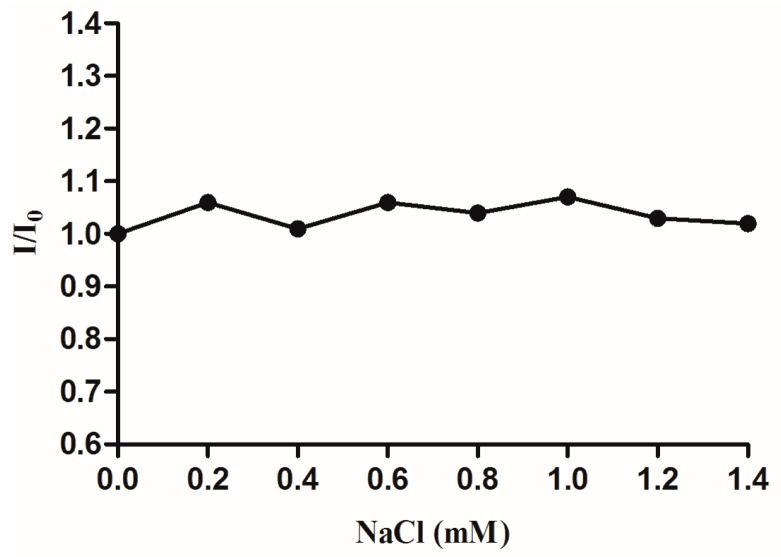
Emission spectra of the DNA-bound complex along with an increasing NaCl concentration.

The effect of ionic strength on the fluorescence intensity of complex-DNA system was also investigated. Since CT-DNA is negatively charged, the DNA is easily surrounded by positively charged sodium ions. If there are electrostatic interactions between ICA-Cu and DNA, the fluorescence intensities of the complex-DNA system would therefore decrease along with the increasing concentration of Na^+^. However, ionic strength experimental results showed that emission intensity of the ICA-Cu-DNA system was nearly constant (Figure 5), with the addition of a series concentration of NaCl. Thus, the interaction mode of ICA-Cu with DNA is not electrostatic interaction.

#### 2.2.4. Quenching Experiment of [Fe(CN)_6_]^3−^

**Figure 6:**
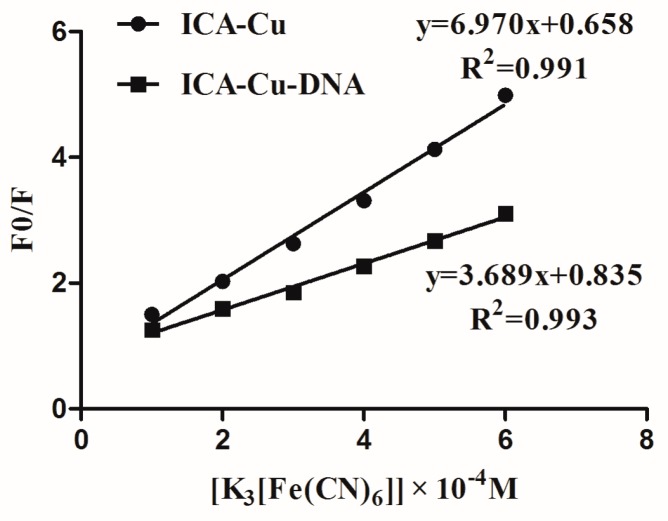
The fluorescence quenching effect of ICA-Cu and ICA-Cu-DNA by K_3_[Fe(CN)_6_] at the emission wavelength of 364 nm.

To clarify the interaction mode of the ICA-Cu complex with DNA, we performed a quenching experiment of [Fe(CN)_6_]^3−^. The Stern-Volmer quenching constant (*K_sv_*) was employed to evaluate the fluorescence quenching efficiency [35]. The fixed concentrations of ICA-Cu or the ICA-Cu-DNA system were mixed with varying concentrations of [Fe(CN)_6_]^3−^ solution, and the fluorescence intensities were then measured. As shown in Figure 6, the plot of free complex ICA-Cu gave *K_sv_* a value of 6.97 × 10^4^ M^−1^. However, the quenching constant decreased in the presence of DNA, and the *K_sv_* value was 3.689 × 10^4^ M^−1^. The great decrease in the *K_sv_* value revealed quite a strong interaction of ICA-Cu with DNA. This agrees with the fact that the fluorescence of the complex was protected after the complex interacted with DNA by an intercalative mode.

#### 2.2.5. Viscosity Measurement

**Figure 7:**
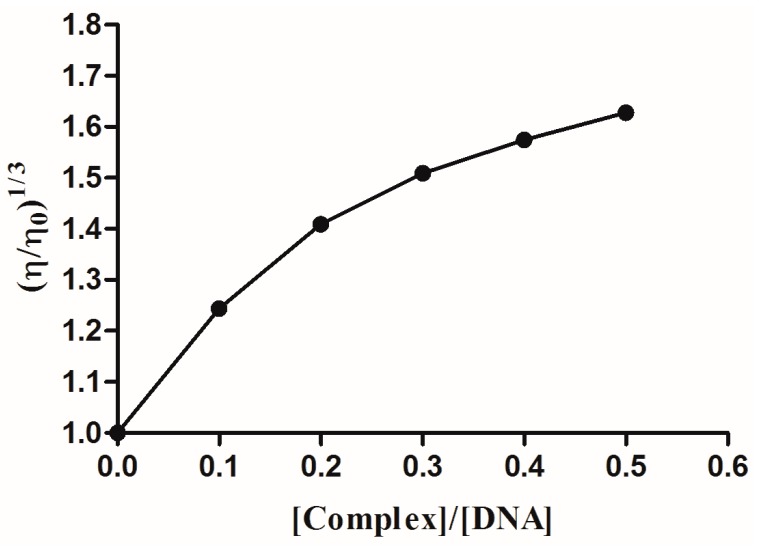
Effect of the increasing amount of ICA-Cu on the relative viscosity of CT-DNA at 298 K. r = 0, 0.1, 0.2, 0.3, 0.4, 0.5 (r = [ICA-Cu]/[DNA]).

Viscosity measurement is one of the available methods to investigate the interacting mode of a complex with DNA, especially an intercalative binding mode [36]. When the temperature of the surroundings is kept constant, the viscosity of the DNA changed with the altering of its length. The relation of relative viscosity (η/η_0_)^1/3^ with DNA length (L/L_0_) meets such an equation; L/L_0_ = (η/η_0_)^1/3^, where L_0_ and L are the lengths of the molecules at the absence and presence of the complex, respectively [37]. Research has shown that there is no change in DNA viscosity when the complex interacts electrostatically with DNA. The binding of the complex to DNA in non-classical intercalative mode led to the decrease of the DNA solution viscosity, which was due to the kink or bend of the DNA helix and the therefore reduced effective length. In contrast, the DNA solution viscosity will increase when the complex binds to DNA in a classical intercalative mode [38]. By plotting the data for (η/η_0_)^1/3^ against [ICA-Cu]/[DNA], we see the results shown in Figure 7. The DNA relative viscosity increases as the ratio of concentration of ICA-Cu and DNA increases. Therefore, the ICA-Cu complex interacts with DNA in the classical intercalation model.

### 2.3. Antioxidant Activity

**Figure 8:**
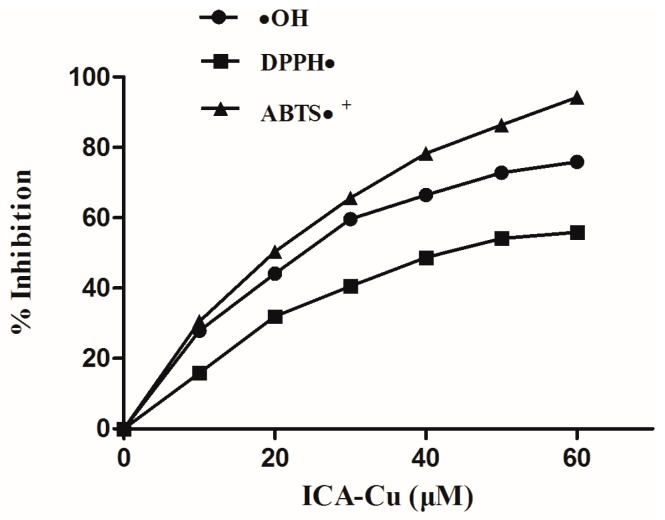
Suppression ratio of ICA-Cu on the •OH, DPPH• and ABTS• free radicals.

Current research has shown that some antioxidants could act as the inducers of DNA damage response, which leads to cell death [39]. Therefore, in present study, we investigated whether the ICA-Cu complex could serve as a potent antioxidant. The scavenging activity of the complex on the DPPH radical, the hydroxyl radical, and the ABTS radical were investigated. The radical was generated in an aqueous media according to the method in the Experimental section. From the results, we can see that, within the range of tested concentration, the average suppression ratios of DPPH, OH, and ABTS increase along with the increase of the complex concentration (Figure 8). The ICA-Cu complex possesses the highest inhibitory activity on the ABTS radical, with 94% inhibition at 60 µM, followed by the OH and DPPH radicals, with the degrees of inhibition being 71% and 56% (Figure 8), respectively.

### 2.4. In Vitro Cytotoxicity Studies 

**Figure 9:**
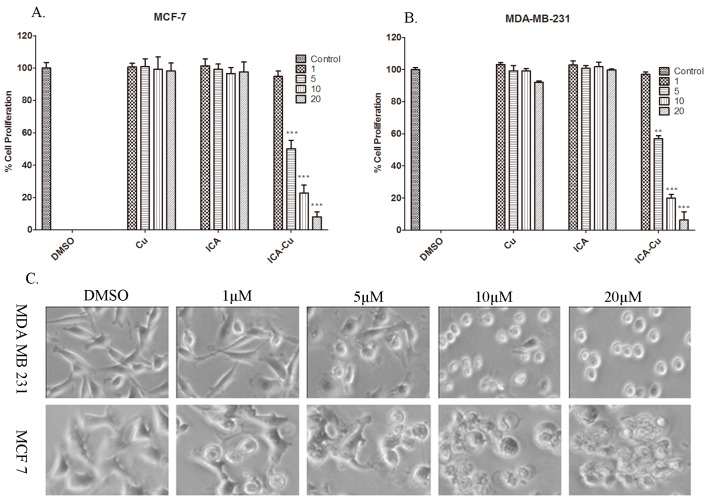
Anticancer activity studies. The MTT assays of MDA-MB-231 (**A**) and MCF-7 cells (**B**) were treated with copper, ICA, and ICA-Cu for 24 h at various concentrations, as indicated. 1% DMSO was used as a control. ** *p* < 0.01 and *** *p* < 0.001, compared to control. The means ± SD of three independent experiments are indicated by the bars; (**C**) the microscopy photographs of MDA-MB-231 and MCF-7 cells treated with ICA-Cu for 24 h, by a magnification of ×400.

To investigate whether ICA-Cu can inhibit cancer cell growth, the human breast cancer cell lines MCF-7 and MDA-MB-231 were treated with 1, 5, 10, and 20 µM ICA, Cu, and ICA-Cu for 24 h (DMSO used as the control), followed by an MTT assay. As shown in Figure 9A,B, ICA-Cu, but not ICA or Cu, was active against the two cell lines. Furthermore, upon increasing the concentration of ICA-Cu, an increase was observed in growth-inhibitory activity and the percentage inhibitions were greater than 90% at 20 µM in both cell lines in a concentration-dependent manner (Figure 9A,B). The IC_50_ values of ICA-Cu on the two cancerous cells were 5.43 and 5.69 µM, respectively. The result appeared that ICA-Cu was toxic to the two human cancer cells. Figure 9C shows cellular morphological changes in two cell lines after treatment with ICA-Cu (with DMSO as a control). The shrunken and rounded up cancer cells were found at concentrations as low as 5 µM (Figure 9C); the morphological changes, along with the increasing concentration of the ICA-Cu complex, confirmed the results of the cytotoxicity studies.

## 3. Experimental Section

### 3.1. Materials and Methods

The Indole-3-acetic acid (ICA) was purchased from J&K Scientific Ltd. (Beijing, China). The Cu(CH_3_COO)_2_·H_2_O, DMSO and KOH were from Aladdin (Beijing, China). The ICA-Cu complex was synthesized in Jining Medical University Pharmacy School (Rizhao, China) and was dissolved in DMSO (50 mM) and stored at room temperature. The CT-DNA was from Shanghai Yuanye Bio-Technology Co., Ltd. (Shanghai, China) and was dissolved in a Tris-NaCl buffer to 2.2 × 10^−4^ M and stored at 4 °C. MTT, DPPH, and ABTS were purchased from Shanghai Yuanye Bio-Technology Co., Ltd. (Shanghai, China). Tris-base and EB were from Solarbio Science & Technology Co., Ltd. (Beijing, China). All other reagents were from Sinopharm Chemical Reagent Co., Ltd. (Shanghai, China).

The electronic absorption spectra were determined on a UV-2450 spectrophotometer (Shimadzu Corp., Tokyo, Japan). The fluorescence intensity measurements were carried out using an F-4600 spectrophotometer (Hitachi Limited, Tokyo, Japan). The infrared spectra were performed on a Nicolet iS50 spectrophotometer (Thermo Fisher Scientific, Waltham, MA, USA) using KBr pellets. The ^1^H-NMR spectra were performed at a Bruker AVANCE III (600-MHz) spectrometer (Bruker, Karlsruhe, Germany). The thermal analyses were recorded on a NETZSCH thermal analyzer (Netzsch, Bavaria, Germany). The elemental analysis (C, H and N) was obtained on a Perkin-Elmer 2400 analyzer (PerkinElmer, Waltham, MA, USA). The cellular changes were recorded using a Zeiss Axiovert 25 microscope (Axiovert, Heidenheim, Germany).

### 3.2. Synthesis of the Complexes

The ligands ICA (0.322 g, 2 mmol) and KOH (0.122, 2 mmol) were dissolved in H_2_O. Cu(CH_3_COO)_2_·H_2_O (0.199 g, 1 mmol) was dissolved in H_2_O, which was added to the above mentioned solution dropwise. The mixture was reacted for 3 h at 40–45 °C to furnish a green precipitate, which was filtered to yield the product. ICA-Cu: [Cu_2_(C_9_H_6_O_2_N)_4_ (H_2_O)_2_]·2H_2_O, FW = 839.75 g·mol^−1^, Yield: 63%. Elemental analysis: calculated (%): C, 51.49; H, 3.84; N, 6.67; found (%): C, 51.57; H, 3.90; N, 6.62. TG analysis: lost 4.34% (calculated 4.29%, 2H_2_O) in 1st step at 25–120 °C; residue 19.75% (calculated 19.05%, CuO). Λ_m_ (S·cm·mol^−1^): 16.52. The IR, UV-Vis, and ^1^H-NMR data of ICA and ICA-Cu are given in Table 1, Table 2 and Table 3.

### 3.3. DNA Binding Experiments 

To understand the binding mode of the ICA-Cu complex with DNA, fluorescence spectra studies were carried out. The concentration of ICA-Cu was set to a constant, while the concentration of CT-DNA was allowed to change in a buffer solution (containing 5 mM Tris and 50 mM NaCl at pH 7.2). Fluorescence excitation and emission spectra were determined after 12 h treatment in a dark place.

The competitive EB binding activities of ICA-Cu were investigated using fluorescent spectrometry. DNA was pretreated with EB in the Tris buffer for 30 min at room temperature. An increasing concentration of the ICA-Cu complex was then added to the mixture (Tris buffer was used as a control) and the fluorescence emission intensity at 592 nm was measured [40].

Ionic strength experiments were carried out in a buffer solution of pH 7.2 (5 mM Tris). DNA was pretreated with the ICA-Cu complex at the same concentration. The fluorescence spectra of the mixture were recorded, along with the increasing concentration of NaCl. 

For the K_3_[Fe(CN)_6_] quenching experiment; in a buffer solution, the complex or the mixture of DNA and the complex with a definite concentration were added to an increasing amount of the K_3_[Fe(CN)_6_] solution and the fluorescence intensity was recorded [41]. 

Viscosity was measured by Ubbelohde viscometer at 25 °C in a constant temperature bath. The concentration of CT-DNA was set to a constant, while the concentration of ICA-Cu changed in the Tris buffer solution. After incubation for 1 h, the flow time of each sample was recorded using a digital stopwatch and measured at least three times. The relative viscosity of DNA was calculated using the relation (η/η_0_)^1/3^, where in η is the viscosity of DNA in the presence of the complexes and η_0_ is the viscosity of DNA without the complexes [37].

### 3.4. Antioxidant Experiments 

The effects of the complexes on radical DPPH were firstly investigated. To a solution of DPPH (0.4 mM) in 75% ethanol was added 1 equal volume of the complexes in ethanol (10, 20, 30, 40, 50, and 60 µM), and ethanol was used as the control. After 30 min in total darkness, the absorbance was determined at 517 nm. The suppression ratio of DPPH• was obtained using the relation (A_0_ − A_i_)/A_0_ × 100%.

Hydroxyl radicals (OH•) were generated through the Fenton-type reaction in an aqueous solution. Reaction mixtures of 10 mL thus obtained contained 4 mL phosphate buffer (pH 7.4), 1.0 mL of 5 mM 1,10-Phenanthroline, 2.0 mL of 2.5 mM FeSO_4_, 1 mL of 0.1% H_2_O_2_, and a series of 2 mL different concentrations of complexes. H_2_O was use as the control. After incubation at 37 °C in a water bath for 60 min, the absorbance at 536 nm was recorded. The scavenging effect for OH• was calculated based on the expression (A_0_ − A_i_)/A_0_ × 100% [42].

For the ABTS•^+^ scavenging assay, the ABTS•^+^ solution was obtained by mixing 0.2 mL ABTS (7.4 mM) with 0.2 mL K_2_S_2_O_8_ (2.6 mM). After incubation for 12 h in darkness at room temperature, the mixture was diluted with 95% ethanol (1:40–50) to adjust the absorbance at 734 nm to 0.70 ± 0.02. To measure the scavenging activity, 0.8 mL of the above-mentioned ABTS•^+^ solution was mixed with 0.2 mL of the sample ethanol solution (with 95% ethanol as control). After 6 min incubation, the absorbance at 734 nm was recorded on the spectrophotometer. The inhibition rates were calculated according to the expression (A_0_ − A_i_)/A_0_ × 100% [43].

### 3.5. In Vitro Anticancer Activity Experiments

The human breast cancer cells MDA-MB-231 and MCF-7 were cultured at 37 °C and 5% CO_2_ in DMEM/F-12(1:1) or RPMI-1640, respectively, with 10% fetal bovine serum (FBS) and 100 units/mL penicillin. MTT assay procedures were used for evaluating the antiproliferative effect of the complex on breast cancer cells MDA-MB-231 and MCF-7. The cells were seeded in a 96-well plate in triplicate and incubated at 37 °C with 5% CO_2_ and grown to 70%–80% confluency. The complexes were dissolved in DMSO and diluted with a culture medium to the indicated concentration, which was added to each well. After treatment for 24 h, the culture medium was removed and the MTT solution (1 mg/mL) was added. After 2 h incubation, the MTT solution was removed and 100 µL DMSO was added to dissolve the metabolized product. The absorbance values were then measured [40]. The cellular morphological analysis was carried out using a Zeiss Axivert 25 microscope (Axiovert, Heidenheim, Germany) with phase contrast.

### 3.6. Statistical Analysis

Statistical analysis was done using Prism 5 and Origin 8.0 (OriginLab, Hampton, MA, USA). The differences between groups were examined through Student’s *t*-test.

## 4. Conclusions

The dinuclear copper(II) complex has been synthesized using indole-2-carboxylic acid as the ligand and its composition and structure have been characterized by elemental analysis, IR, UV-Vis, ^1^H-NMR, TG analysis, and molar conductance, and its molecular formula was [Cu_2_(C_9_H_6_O_2_N)_4_(H_2_O)_2_]·2H_2_O. Furthermore, the biological and anticancer activities of the complex were investigated. The results of fluorescence spectrometry, ionic strength, and the viscosity method indicated that ICA-Cu possessed a high binding ability to DNA in an intercalative mode. The EB-competitive DNA-binding studies suggested that ICA-Cu could displace EB from the EB-DNA complex, and thus make the fluorescence intensity of the EB-DNA complex decrease sharply. The quenching constant *K_SV_* value was 3.99 × 10^4^ M^−1^. The quenching experiment of [Fe(CN)_6_]^3−^ showed that free complex ICA-Cu gave a *K_sv_* value of 6.97 × 10^4^ M^−1^, and the *K_sv_* value decreased to 3.689 × 10^4^ M^−1^ in the presence of DNA, suggesting that fluorescence of the complex was protected after the complex interacted with DNA through an intercalative mode.

It may be clearly seen from the results of the antioxidant activities that, within the range of the tested concentration, the average suppression ratios of DPPH, OH, and ABTS increased along with the increase in concentration of the complex. The ICA-Cu possessed the highest inhibitory effect for the ABTS radical, with 94% inhibition at 60 µM, followed by the OH and DPPH radicals, with the degrees of inhibition being 71% and 56%, respectively.

The in vitro cytotoxicity of ICA-Cu against the human breast cancer cell lines MDA-MB-231 and MCF-7 was investigated by an MTT assay and cellular morphological analysis. Upon increasing the concentration of ICA-Cu, an increase was observed in growth-inhibitory activity and the percentages of inhibition were higher than 90% at 20 µM in both cell lines. Also, cellular morphological changes in the two cell lines confirmed the cytotoxicity results. These results clearly demonstrated that the ICA-Cu complex may be a promising lead compound for anticancer drug development.

## Figures and Tables

**Table 1:** Main IR data for the ICA and the ICA-Cu complex (cm^−1^).

Ligand/Complex	*ν*_-NH-_	*ν_as_*_(COO^−^)_	*ν_s_*_(COO^−^)_	*ν*_Cu-O_
ICA	3355.75	1696.19	1200.91	-
ICA-Cu	3437.61	1510.92	1344.34	484.64

**Table 2:** UV-Vis spectra data of the ICA and the ICA-Cu complex (nm).

Ligand/Complex	λ_max_
ICA	292
ICA-Cu	298

**Table 3:** ^1^H-NMR spectra data of the ICA and the ICA-Cu complex (ppm).

Ligand/Complex	H-3	H-4	H-5	H-6	H-7	-COOH	-NH-
ICA	7.252 (m)	7.649 (m)	7.051 (m)	7.238 (m)	7.442 (m)	11.753 (s)	12.943 (s)
ICA-Cu	7.229 (m)	7.519 (m)	6.082 (m)	7.042 (m)	7.388 (m)	-	14.476 (s)
